# Reduced Prepulse Inhibition as a Biomarker of Schizophrenia

**DOI:** 10.3389/fnbeh.2016.00202

**Published:** 2016-10-18

**Authors:** Auxiliadora Mena, Juan C. Ruiz-Salas, Andrea Puentes, Inmaculada Dorado, Miguel Ruiz-Veguilla, Luis G. De la Casa

**Affiliations:** ^1^Department of Experimental Psychology, University of SevilleSeville, Spain; ^2^Neurosciences Institute, El Bosque UniversityBogotá, Colombia; ^3^Institute of Biomedicine, University Hospital Virgen del RocíoSeville, Spain

**Keywords:** startle, prepulse inhibition, habituation, schizophrenia, endophenotype

## Abstract

The startle response is composed by a set of reflex behaviors intended to prepare the organism to face a potentially relevant stimulus. This response can be modulated by several factors as, for example, repeated presentations of the stimulus (startle habituation), or by previous presentation of a weak stimulus (Prepulse Inhibition [PPI]). Both phenomena appear disrupted in schizophrenia that is thought to reflect an alteration in dopaminergic and glutamatergic neurotransmission. In this paper we analyze whether the reported deficits are indicating a transient effect restricted to the acute phase of the disease, or if it reflects a more general biomarker or endophenotype of the disorder. To this end, we measured startle responses in the same set of thirteen schizophrenia patients with a cross-sectional design at two periods: 5 days after hospital admission and 3 months after discharge. The results showed that both startle habituation and PPI were impaired in the schizophrenia patients at the acute stage as compared to a control group composed by 13 healthy participants, and that PPI but not startle habituation remained disrupted when registered 3 months after the discharge. These data point to the consideration of PPI, but not startle habituation, as a schizophrenia biomarker.

## Introduction

The information processing that is carried out by organisms with complex nervous systems begins with a set of automatic processes of a preattentional nature that usually occur during the first 100 ms after the onset of the stimuli (Ellenbroek, 2004). These processes trigger various response mechanisms, one category of which is the startle response, a neuronal mechanism related to attention that varies along a continuum from deep sleep to intense excitement (Dawson et al., 1999). The startle response includes a reflex including a startle and/or orienting response that is evoked by a stimulus of sufficient intensity (Yeomans et al., 2002) and it reflects the activation of motor tracts in the brainstem, particularly the bulbopontine reticular formation (Brown et al., 1991). The basic physiological pattern of the acoustic startle reflex is mediated by a circuit involving the statoacoustic nerve, the ventral cochlear nucleus, lateral lemniscus, nucleus reticularis pontis caudalis, spinal interneuron, motor neurons of the anterior Horn, peripheral nerves, and muscles (Davis et al., 1982; Lee et al., 1996).

The adaptive role of the startle reflex response is clear when we consider its plasticity in response to various circumstances, which can lead to either an increase or decrease in its intensity. Thus, for example, the startle response increases when the organism is in a state of emotional activation due to a process of sensitization (Thompson and Glanzman, 1976) or decreases with repeated presentations of a stimulus due to the process of habituation (Tighe and Leaton, 1976). A decline in the startle response is also observed when a stimulus of low intensity precedes a more intense stimulus, a phenomenon known as Prepulse Inhibition (PPI; Hoffman and Searle, 1968; Graham and Murray, 1977).

The study of the startle response (and its plasticity) within the field of schizophrenia has provided a wealth of information concerning the possible biological and psychological processes underlying this disorder (e.g., Braff et al., 2001a; Ludewig et al., 2002). On the one hand, numerous studies focusing on the habituation of the startle response in schizophrenia patients have led to apparently contradictory results, since some studies have identified a decrease in habituation of this response in patients with schizophrenia (Geyer and Braff, 1982; Braff et al., 1992; Parwani et al., 2000), whereas in other work, habituation appears to remain intact (Braff et al., 1999; Kumari et al., 1999, 2002; Cadenhead et al., 2000).

Some authors have suggested that, given that PPI can reflect the ability to regulate the amount of sensory information that is processed at all times, and that it plays a key role in filtering relevant and irrelevant sensory information, alteration of this effect in schizophrenia patients could be a cause of the overload of sensory information that is characteristic of this disease (e.g., Perry and Braff, 1994). Indeed, a decline in PPI in schizophrenia patients compared to healthy participants has been consistently demonstrated in the literature (e.g., Braff et al., 1992, 1999; Grillon et al., 1992; Kumari et al., 1999; Parwani et al., 2000; Takahashi et al., 2008; Moriwaki et al., 2009), and it has thus been proposed as a biomarker of the disease (e.g., Walters and Owen, 2007; Light and Swerdlow, 2014). In particular, Gottesman and Gould (2003) have proposed that PPI may be considered a schizophrenia endophenotype, that is, a subtype of biomarker that reflects the link between genetic and clinical expression (see, also, Turetsky et al., 2007; Light et al., 2012).

The importance of identifying biomarkers of health becomes apparent if we consider these to be objective indicators that can provide information on the origin of the disorder, the prognosis, and possible response to treatment (e.g., Light and Swerdlow, 2014). The notion of PPI as a biomarker for sensorimotor gating in schizophrenia is supported not only by the changes observed in schizophrenia patients, but also the increased heritability observed in the decline in PPI in families with high genetic vulnerability for schizophrenia (Hasenkamp et al., 2010; Greenwood et al., 2016). Thus, for instance, Anokhin et al. (2003) reported a 50% of PPI variance explained by genetic factors in a sample of 142 female twins. Further, decreases in PPI have been observed in asymptomatic first-degree relatives of schizophrenia patients (Cadenhead et al., 2000), in patients with schizotypal personality disorder (Cadenhead et al., 1993), and in healthy subjects that score highly on tests designed to assess propensity toward psychosis (Kumari et al., 2008). Finally, the stability across time of PPI deficits in schizophrenia that is evident even before the symptoms appear and that remains stable for as long as 2 years (Ziermans et al., 2011), or the growing evidence on common genetic factors of PPI abnormalities and schizophrenia (e.g., Roussos et al., 2016), support the notion of PPI as an endophenotype of schizophrenia.

A critical aspect for determining the possible role of PPI as a biomarker of the schizophrenic disorder is related to the long-term stability of the PPI deficit in schizophrenia patients. Evidence relating to this issue is still relatively scarce and sometimes seemingly contradictory, since some researchers have found that the PPI deficit remains constant over time (Ludewig et al., 2002; Mackeprang et al., 2002; Duncan et al., 2003; Düring et al., 2014), whilst in other studies, however, it has been found that deficient PPI in schizophrenia patients returned to normal levels when measurements were taken in the chronic phase of the disorder (e.g., Meincke et al., 2004b; Quednow et al., 2006; Minassian et al., 2007; Martinez-Gras et al., 2009; Aggernaes et al., 2010; Hammer et al., 2011). Details of longitudinal studies examining PPI in schizophrenia are listed in **Table 1**. As it is evident from the findings described in **Table 1**, deficits in PPI were consistently observed when PPI was initially registered, irrespective of the stage of the disease (chronic or acute). However, when PPI was retested in a follow up, it remained disrupted in four reports but it was normalized in six experiments.

**Table 1:** Longitudinal studies of PPI in schizophrenia.

	Participants	Test–Retest	Treatment (Schizophrenia sample)	PPI at T1	PPI at T2 (and T3)
Ludewig et al., 2002	Schizophrenia: 19	T1: Chronic	Mixed medication	Reduced^a^	Reduced
	Control: 24	T2: 4 weeks			
		T3: 8 weeks			
Mackeprang et al., 2002	Schizophrenia: 20	T1: Acute	Typical vs. Atypical	Reduced	Reduced^b^
	Control: 20	T2: 12 weeks			
Duncan et al., 2003	Schizophrenia: 15	T1: Chronic	Mixed medication	Reduced	Reduced
	Control: No	T2: 12 weeks			
Meincke et al., 2004b	Schizophrenia: 36	T1: Acute	Mixed medication	Reduced^c^	Normalized
	Control: 18	T2: 2–3 weeks			
Quednow et al., 2006	Schizophrenia: 28	T1: Acute	Amisulpride vs. Olanzapine	Reduced	Normalized^d^
	Control: 18	T2: 4 weeks			
		T3: 8 weeks			
Minassian et al., 2007	Schizophrenia: 23	T1: Acute	Mixed medication	Reduced	Normalized
	Control: 20	T2: 2 weeks			
Martinez-Gras et al., 2009	Schizophrenia: 45	T1: Chronic	Zuclopenthixol (T1) vs. risperidone (T29)	Reduced	Normalized
	Control: 36	T2: 12 weeks			
Aggernaes et al., 2010	Schizophrenia: 16	T1: Acute	All quetiapine	Reduced^e^	Normalized
	Control: 14	T2: 24 weeks			
Hammer et al., 2011	Schizophrenia: 13	T1: Acute	Mixed medication	Reduced^f^	Normalized^f^
	Control: 17	T2: 6 year			
Düring et al., 2014	Schizophrenia: 51, 15	T1: Acute	All amisulpride	Reduced	Reduced
	Control: 47	T2: 2 weeks			
		T3: 6 weeks			


*^a^PPI was reduced only at the 86 dB/60 ms condition. ^b^PPI was restored irrespective of treatment with typical or atypical. ^c^PPI was reduced only at the 73 dB/100 ms condition. ^d^PPI was restored irrespective of treatment with amisulpride or olanzapine. ^e^PPI was reduced only for the males sample in the 85 Db/60 ms condition. ^f^PPI was reduced and normalized when the lead interval was 60 ms, but not with 30 or 120 ms.*

On the basis of these considerations, the current paper has three chief aims. The first is to confirm the attenuation of PPI in patients diagnosed with schizophrenia compared with a control sample of healthy participants. The second is to analyze the possible impairment in habituation to the startle response in schizophrenia patients. Finally, we aim to evaluate the persistence and stability of these deficits in schizophrenia patients (both in PPI and habituation of the startle response) by conducting a retest 3 months after discharge. We expect to find attenuation of both habituation of the startle response and PPI when comparing the results of schizophrenia patients (in the acute phase of the disease) with healthy controls. Moreover, if, as suggested by the literature, the reduction in PPI is a biomarker for schizophrenia and therefore remains stable over time, it is expected that this PPI deficit would appear in the acute phase and would remain intact 3 months after discharge of the patients.

## Materials and Methods

### Participants

Twenty-eight patients with a DSM-IV diagnosis of paranoid schizophrenia, and 28 healthy controls matched for gender and age, were initially enrolled at the inpatient services at the Virgen del Rocío University Hospital, Seville (Spain). All patients had been diagnosed by an experienced psychiatrist, and the controls were screened for history of mental illness, drug and alcohol abuse, or regular medical prescription. Of the initial sample, 15 patients refused to participate in the follow up assessment. Therefore, 13 schizophrenia patients (10 male, and three female; mean age 37.31 years, range 21–61 years), and their correspondent thirteen healthy controls (nine males and four females; mean age 36.77, range; 21–60 years) were included in the final sample. All patients had been admitted for psychotic decompensation or psychotic episode. The startle testing for the schizophrenia patients was conducted 3–8 days after admission to hospital (acute stage) and 3 months after the discharge (chronic stage), and mean number of days elapsed from the test at the acute stage to discharge was 13.69 (*SD* = 9.88). Clinical and demographic characteristics of the participants, including Positive and Negative Syndrome Scale (PANSS) scores and medication, are presented in **Table 2**. All patients were receiving atypical antipsychotics before their inclusion in this experiment, with doses between 300 and 750 mg/day (Chlorpromazine equivalent), mean = 403.57 mg/day during the acute period, and doses between 250–750 mg/day, mean = 349 mg/day during the chronic period. PANNS General and Total scores were significantly lower for the retest (chronic) as compared to the test (acute) period. After being informed of the type of stimulation used in the experiment, all subjects gave signed informed consent to participate before to start the experimental manipulations in accordance with the Declaration of Helsinki. This study was carried out in accordance with the recommendations of University of Seville’s ethical committee.

**Table 2:** Demographic and clinical characteristics of the participants.

	Patients (acute)	Patients (chronic)	Controls
Male/Female	10/3	10/3	9/4
Age (years)	37.31 (13.31)		36.77 (12.38)
Age at onset (years)	25 (8.2)		
Number of episodes	4.17 (2.99)		
Smokers	10		5
PANSS-Positive	22.08 (5.37)	12.20 (3.88)	
PANSS-Negative	16.42 (6.16)	15.20 (5.79)	
PANSS-General	33.00 (9.66)	24.10 (8.20)^∗∗^	
PANSS-Total	71.5 (15.88)	51.50 (16.46)^∗^

	**Medication**	

	3 quetiapine	3 quetiapine
	4 risperidone	2 risperidone
	4 paliperidone	3 paliperidone
	1 aripiprazole	1 aripiprazole
	1 olanzapine	2 olanzapine
		1 clozapine
		1 untreated


*Standard Deviations appear between brackets. PANSS scores were compared at chronic vs. acute period (*t*-tests for related samples, one-tailed): ^∗^*p* < 0.05; ^∗∗^*p* < 0.001.*

### Procedure

Acoustic stimuli were delivered binaurally using adjustable headphones (Sony model MDR-V50), connected to a MP150 control module (Biopac Systems Inc., Goleta, CA, USA). The signal was sent with a high sampling rate of 50 kHz. The prepulse and the pulse stimulus consisted of a 80 dB (A) and 106 dB (A) white noise with instantaneous rise time, lasting for 20 and 50 ms, respectively. A background noise (white noise, 70 dB) was presented during the entire duration of the experiment. Sound calibration was completed prior to record data for each participant using a Sound Level Meter PCE-999.

The experiment was conducted in an isolated room. For all auditory trials, the ITI was 15 s (+/- 5 s) and the lead interval in prepulse-pulse trials was 40, 60, or 80 ms. After a 120 s adaptation period to the background noise, four pulses were presented in order to establish the baseline response to the auditory stimuli. Next, it started the experimental stage composed by six blocks of four trials each that were presented at random for each participant. Each block included two pulse-alone and two prepulse-pulse trials. For two of the blocks the lead interval for the prepulse-pulse trials was 40 ms, for other two blocks it was 60 ms, and for the last two blocks it was 80 ms. Finally, four pulse-alone trials were presented in order to check habituation of the startle response.

Electromyographic (EMG) activity of the orbicularis oculi muscle was recorded using three Ag/AgCl electrodes (EL250; Biopac Systems) positioned according to the guidelines recommended by Blumenthal et al. (2005). Specifically, after cleaning the participant’s skin, conductive gel was applied to the electrodes before placing two of them approximately 1 cm below the right eye to record the EMG activity of the orbicularis oculi muscle. The third electrode was placed on the forehead to detect the general level of electrical activity. Raw signals were amplified (×2000) and filtered using a passband of 10–500 Hz (EMG100C amplifier; Biopac Systems). AcqKnowledge software (4.0, Biopac Systems) was used to interface a MP150 control module (Biopac Systems) via a cross-over cable and sampled at 2 kHz. Blink responses were considered valid when they occurred between 21 and 120 ms after the pulse alone stimulus. Trials were discarded when excessive EMG activity was observed during the first 20 ms of recording. Less than 5% were discarded using these parameters.

## Results

A preliminary analysis was conducted to evaluate the normality of distributions of Startle and PPI scores (Shapiro–Wilk tests, *p* < 0.05). Only startle magnitude to the Pulse-alone trials (Pre- and Post-Experimental) for the acute Schizophrenia patients did not follow the normal distribution. Therefore, those data were explored with non-parametric test. The remaining analyses were performed using ANOVAs.

### Between Subjects Comparisons (Groups: Acute Schizophrenia vs. Control)

#### Analysis of Startle Magnitude to the Pulse-alone Trials

**FIGURE 1:**
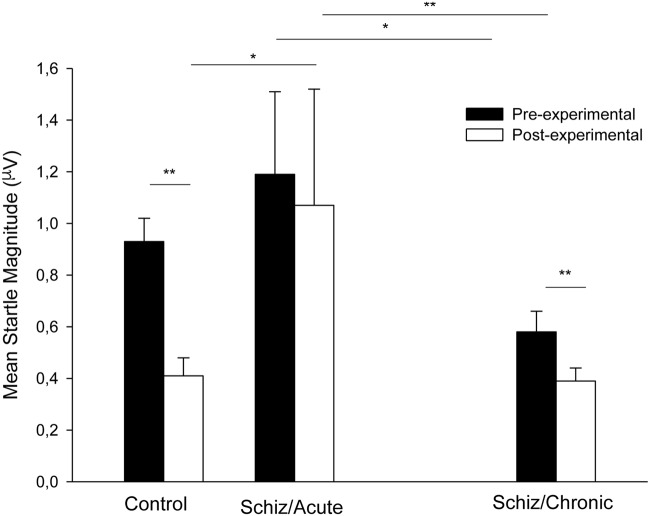
**Mean startle magnitude collapsed across trials for the Pulse-alone trials presented before and after prepulse measurement registered for the control participants (left section), for the schizophrenia patients tested 3–7 days after admission (middle section), and for the chronic patients tested 3 months after discharge (right section).** Error bars represent standard error of the mean. ^∗^*P* < 0.05; ^∗∗^*p* < 0.01 (see text for statistical details).

**Figure 1** shows mean startle magnitude for the pre- and post-experimental Pulse-alone trials (collapsed) registered during the acute phase for the schizophrenia patients (middle section) and the control participants (left section). As can be seen in the **Figure 1**, startle habituation (indicated by a decrease in startle magnitude from the pre- to the post-experimental period) was only evident for the control group.

Since startle response to the Pre- and Post-experimental Pulse-alone trials registered at the acute stage for schizophrenia patients were not normally distributed, we used non-parametric Mann–Whitney (independent samples) and Wilcoxon Signed-ranks tests (related samples) to explore this variable. Startle magnitude was similar for the Control and Schizophrenia participants at the Pre-experimental period (*U* = 82.00, *p* > 0.89), but the startle magnitude was more intense for the Schizophrenia patients than for the Control participants at the post-experimental period (*U* = 42.00, *p* < 0.05, respectively). Startle magnitude at Pre-experimental period was significantly more intense as compared to that registered at Post-experimental period for the Control participants (*Z* = 3.18, *p* < 0.01), but not for the Schizophrenia patients (*Z* = 1.15, *p* > 0.24). These results reveal that startle magnitude was reduced across trials for the control participants reflecting startle habituation, but habituation did not occur for the acute schizophrenia patients.

#### Analyses of Percent PPI

Several studies have suggested that percent PPI is less contaminated by individual differences than raw PPI (Hawk and Cook, 2000). Accordingly, mean startle magnitudes for pulse and prepulse-pulse trials were converted into percent PPI, calculated as 100 × (Average startle to the pulse – Average startle to the prepulse-pulse)/Average startle to the pulse. Since it has been reported that differences in reactivity to pulse-alone stimulus can influence percent PPI (e.g., Csomor et al., 2008), we conducted a preliminary one-way ANOVA on mean startle intensity as a function of Group that revealed the absence of differences between the Control and the Acute Groups, *F*(1,24) < 1.

**FIGURE 2:**
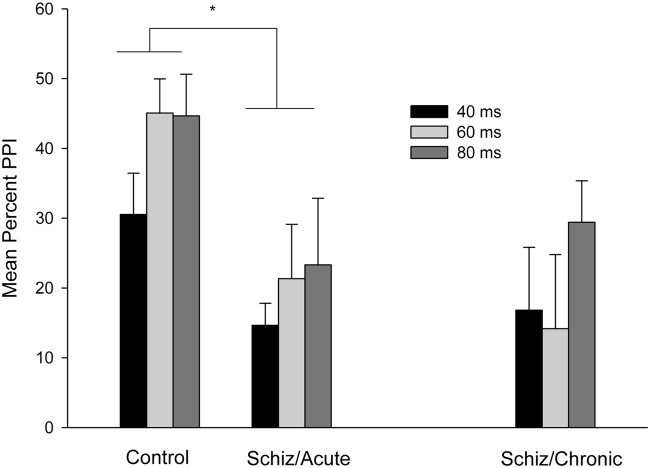
**Mean percent PPI as a function of Prepulse-Pulse interval (40, 60, or 80 ms) for the control participants (left section), for the schizophrenia patients tested 3–7 days after admission (middle section), and for the chronic patients tested 3 months after discharge (right section).** Error bars represent standard error of the means. ^∗^*P* < 0.05 (see text for statistical details).

**Figure 2** depicts mean percent PPI as a function of the 40, 60, and 80 ms lead interval conditions for control and schizophrenia participants (PPI for patients registered at acute and chronic periods). Attending to the between-subject comparison involving PPI for Control (left section) vs. Acute schizophrenia (middle section) participants that is depicted in **Figure 2**, PPI increased across lead intervals, with higher PPI values for the 80 and 60 ms, as compared to the 40 ms condition. In addition, the PPI effect was reduced in the Acute schizophrenia patients as compared to the control groups.

These impressions were confirmed by the statistical analyses. A mixed 3 × 2 ANOVA (Lead interval: 40 vs. 60 vs. 80 ms × Group: Acute vs. Control) conducted on mean percent PPI revealed significant main effects of Lead and Groups, *F*(2,48) = 3.87; *p* < 0.05, and *F*(1,24) = 7.18; *p* < 0.05, respectively. The two-way interaction was non-significant, *F*(2,48) < 1. The main effect of Groups reflects a higher overall percent PPI for the Control (Mean = 40.09%, *SD* = 18.76) as compared to the Acute Group (Mean = 19.76%, SD = 19.91). As for the significant effect of Lead interval, *t*-tests for related samples (*p <* 0.05, one-tailed) were conducted on mean percent PPI. The analysis revealed an overall higher PPI for the 60 and 80 ms, (Mean = 33.21%, *SD* = 25.97, and Mean = 33.99%, *SD* = 30.16, respectively) as compared to the 40 ms condition (Mean = 22.59%, *SD* = 18.66).

### Within-Subject Comparisons (Acute vs. Chronic)

#### Analysis of Startle Magnitude to the Pulse-alone Trials

**Figure 1** (right section) depicts mean startle magnitude for the four Pulse-alone trials (collapsed) presented before and after prepulse measurement recorded for the schizophrenia participants during the chronic period (3 months after the release). As can be seen in the figure, the startle response magnitude and the habituation process were normalized at the chronic period as compared to that observed at the acute period (middle section of **Figure 1**).

Non-parametric Wilcoxon Signed-ranks tests (related samples) revealed higher startle magnitude during the acute as compared to the chronic stage both for the Pre- and the Post-experimental periods (*Z* = 2.27, *p* < 0.05, and *Z* = 2.69, *p* < 0.01, respectively). Interestingly, habituation of startle, namely the reduction of startle intensity across Pulse-alone presentations that did not appear at the acute period (*Z* = 1.15, *p* > 0.24) was significant when registered at the chronic period (*Z* = 2.69, *p* < 0.01).

#### Analyses of Percent PPI

**Figure 2** (middle vs. right section) depicts mean percent of PPI for schizophrenia patients as a function of the 40, 60, and 80 ms lead interval conditions registered at the acute and chronic periods. As can be seen in the figure, PPI remained unchanged at the acute as compared to the chronic period for all lead interval conditions. Consistently with this impression, a mixed 3 × 2 ANCOVA conducted on mean percent PPI (Lead interval: 40 vs. 60 vs. 80 ms × Group: Acute vs. Chronic, both factors within-subject; chlorpromazine equivalent dose and startle magnitude for the acute and chronic time points were included as covariates) revealed non-significant main effects nor interactions (all *p*s > 0.29).

### Correlations between PANSS Scores and PPI Magnitude

In order to analyze the relationship of symptoms in acute and chronic stages with PPI magnitude, Pearson R correlations were calculated between mean PPI for the 40, 60, and 80 ms lead intervals and PANSS scores. There were no significant correlations of PPI with any of the sub-scales of PANSS or total PANSS score (all *p*s > 0.08).

## Discussion

Although taken with caution due to the small sample size^1^, the analyses that compare the results of healthy control participants with those obtained in acute schizophrenia patients have revealed significant differences in both habituation of the startle response and intensity of PPI. More specifically, participants without pathologies showed normal habituation of the startle response after repeated presentations of an intense auditory stimulus (pulse), while for patients in acute phase the startle response remained at the same intensity despite repeated presentations of the stimulus. Some authors propose that this absence of habituation of the startle response could serve as a trait marker of schizophrenic psychoses (e.g., Meincke et al., 2004a). With respect to the effect on PPI, our results reproduce those obtained in the previous literature, since we observed a significant reduction in the average percentage of PPI in acute schizophrenia patients when compared with that observed in the healthy control group. This data allows us to confirm that, at least with the parameters used in our experiment, PPI appears to be impaired in schizophrenia patients, a result that has been well documented in previous literature (e.g., Meincke et al., 2004b; Quednow et al., 2006; Minassian et al., 2007; Martinez-Gras et al., 2009; Aggernaes et al., 2010; Hammer et al., 2011).

With regard to the results reported in schizophrenia patients 3 months after discharge, once the symptoms had decreased, a recovery of the process of habituation of the startle response was observed, while the impairment in PPI remained unchanged from that recorded in the acute stage of the disease. This conclusion could be compromised by the lack of follow-up data from control participants, since the level of habituation might still be impaired in patients relative to controls at retest, and PPI might have been also reduced in the control sample after 3 months. However, these possibilities are unlikely considering that the improvement in startle habituation in schizophrenia is based on a significant interaction between two within-subject factors (Period: Pre- vs. Post-experimental and Schizophrenia state: Acute vs. Chronic) supporting that the change of startle habituation from the acute to the chronic period is a relevant result by itself, independently of any hypothetical data from control participants at follow-up. In addition, and regarding the PPI result, the well-established high test–retest reliability of PPI in healthy humans and schizophrenia patients (e.g., Abel et al., 1998; Flaten, 2002), allow us to anticipate that PPI would have remained stable at retest for the control sample. An additional potential factor that could have affected to the data from the schizophrenia patients is related to the elevated proportion of smokers in our sample (close to 80%). However, it has been demonstrated that nicotine increases PPI in healthy humans (e.g., Della Casa et al., 1998), in schizophrenia patients (e.g., Hong et al., 2008), and in rodents (e.g., Pinnock et al., 2015), but our data indicates that PPI was reduced for the Schizophrenia patient’s sample. Therefore, we can discard an effect of the smoking status on our results. There are also some other limitations due to the lack of experimental control on several factors that have demonstrated a relationship with startle and PPI magnitude. Thus, we did not get SCID confirmation of diagnoses, participants’ hearing acuity was not evaluated before the experimental treatment, and time of menstrual phase for female participants was not controlled. Especially, the last factor has been related to PPI intensity, since it is reduced in the luteal as compared to the follicular phase of the menstrual cycle (Swerdlow et al., 1997; Jovanovic et al., 2004). However, given the low number of female participants in our sample (three in the schizophrenia patients group, and four in the control group), we anticipate that the effect of such variable may not have affected the results. In spite of the mentioned limitations, our findings suggest that an attenuation of PPI, but not the absence of habituation of the startle response, may be considered as a biomarker of the schizophrenic disorder.

The relevance of identifying biomarkers in the field of mental illness, especially in the case of schizophrenic disorder, has recently been highlighted by Light and Swerdlow (2014), who note that PPI is one of the processes that meets the criteria identified by two panels of experts that have gathered in the past decade to identify the most appropriate therapies for the treatment of cognitive processes in schizophrenia patients: The Measurement and Treatment Research to Improve Cognition in Schizophrenia (MATRICS; Green et al., 2004), and the Cognitive Neuroscience Treatment Research to Improve Cognition in Schizophrenia (CNTRICS; Carter et al., 2008). More specifically, the criteria indicated by both initiatives as being necessary to accept a measure as being a valid neurophysiologic biomarker include, among other things, stability over time, which is independent of state-related changes, the possibility of conducting repeated measures, reactivity to pharmacological treatment which can be reproduced in an animal model, and to be associated with neural circuits and clearly identified cognitive mechanisms (e.g., Green et al., 2009).

Of those criteria, our results provide evidence for the stability of the deficit in PPI across different phases of schizophrenia, an aspect of particular importance if we consider the lack of conclusive evidence in previous literature. In particular, while some studies have shown that disrupted PPI remains intact independently of whether it is evaluated in schizophrenia patients treated with traditional antipsychotics (Mackeprang et al., 2002; Kunugi et al., 2007) or with atypical antipsychotics (Mackeprang et al., 2002; Duncan et al., 2003), other work has shown a recovery of PPI in schizophrenia after treatment with atypical antipsychotics (Kumari et al., 1999; Leumann et al., 2002; Oranje et al., 2002; Martinez-Gras et al., 2009). In our sample, all patients were prescribed with atypical antipsychotics, but the reduced PPI observed both in the acute and the chronic periods indicates that the drug treatment was ineffective to improve PPI. This result contrasts with some previous reports indicating that patients medicated with the atypicals risperidone (Mackeprang et al., 2002), clozapine (Oranje et al., 2002; Kunugi et al., 2007) or long-acting risperidone (Martinez-Gras et al., 2009) showed an improvement in sensorimotor gating. It is worth noting, however, that in our sample only four patients received risperidone at the acute stage (two at the chronic period), and only one was prescribed with clozapine at the chronic period, that can have reduced the impact of medication on PPI normalization. Future research should focus on differential effects of specific antipsychotic medication of PPI taking advantage from within-subjects longitudinal designs.

As we noted in the introduction, PPI has been proposed as a model to detect differences in the processing of information related to some structures and systems that are considered essential in the development of schizophrenia, such as the mesolimbic system or dopaminergic activity (e.g., Swerdlow et al., 2001). From a cognitive standpoint, there is abundant evidence that patients with schizophrenia have significant limitations both in voluntary control of attention and control of the automatic, preattentional processes that are responsible for limiting access to irrelevant stimuli in order to process subsequent sensorimotor integration (Braff et al., 1978). This process is essential for adequate adaptation, since it allows selection of the most important elements of the environment and prevents overload of sensory information that could contribute to disorders such as those that characterize the occurrence of schizophrenia.

In our work we have provided direct evidence related to the stability of PPI by collecting data at two different time points separated by an interval of 3 months, using a test–retest methodology. In spite of the difficulties in persuading patients to agree to participate again in the collection of data after being discharged, (which restricted the size of our participant sample), we believe that the results are particularly relevant since for the most part, the evidence available in the literature has been obtained by indirect means such as comparing patient samples with healthy participants (see, for reviews, Braff et al., 2001b; Swerdlow et al., 2008).

## Author Contributions

LD, AM, and MR-V designed the experiment; AM, JR-S, AP, and ID performed the experiment; LD, AM, and JR-S analyzed data; LD, AM, and MR-V wrote the manuscript.

## Conflict of Interest Statement

The authors declare that the research was conducted in the absence of any commercial or financial relationships that could be construed as a potential conflict of interest.

## Footnotes

1 In spite of the small sample size, the results can benefit from the specific *a priori* hypotheses we have proposed, and the advantages of a within-subject design, with lower error variability than similar between-subjects designs.
